# Effective Excess Noise Suppression in Continuous-Variable Quantum Key Distribution through Carrier Frequency Switching

**DOI:** 10.3390/e25091286

**Published:** 2023-08-31

**Authors:** Jing Dong, Tao Wang, Zhuxuan He, Yueer Shi, Lang Li, Peng Huang, Guihua Zeng

**Affiliations:** 1 State Key Laboratory of Advanced Optical Communication Systems and Networks, Center of Quantum Sensing and Information Processing, Shanghai Jiao Tong University, Shanghai 200240, China; 2 Shanghai Research Center for Quantum Sciences, Shanghai 201315, China

**Keywords:** continuous-variable, quantum key distribution, frequency switching, noise suppression

## Abstract

Continuous-variable quantum key distribution (CV-QKD) is a promising protocol that can be easily integrated with classical optical communication systems. However, in the case of quantum-classical co-transmissions, such as dense wavelength division multiplexing with classical channels and time division multiplexing with large-power classical signal, a quantum signal is more susceptible to crosstalk caused by a classical signal, leading to signal distortion and key distribution performance reduction. To address this issue, we propose a noise-suppression scheme based on carrier frequency switching (CFS) that can effectively mitigate the influence of large-power random noise on the weak coherent state. In this noise-suppression scheme, a minimum-value window of the channel’s noise power spectrum is searched for and the transmission signal frequency spectrum shifts to the corresponding frequency to avoid large-power channel noise. A digital filter is also utilized to filter out most of the channel noise. Simulation results show that compared to the traditional fixed carrier frequency scheme, the proposed noise-suppression scheme can reduce the excess noise to 1.8%, and the secret key rate can be increased by 1.43 to 2.86 times at different distances. This noise-suppression scheme is expected to be applied in scenarios like quantum–classical co-transmission and multi-QKD co-transmission to provide noise-suppression solutions.

## 1. Introduction

Quantum key distribution (QKD) is a secure key distribution protocol based on the principles of quantum mechanics. It uses the properties of quantum entanglement and the non-orthogonal basis to transmit and share cryptographic keys. Due to the inherent properties of quantum mechanics, any attempt to eavesdrop on the key will be detected, ensuring the security of the key distribution. Continuous-variable quantum key distribution (CV-QKD) is a significant type of QKD that can be easily integrated with classical optical communication systems. CV-QKD employs classical photoelectric components to generate and modulate coherent states at the transmitter, and detects the coherent states using coherent detection at the receiver. These features make CV-QKD an attractive candidate for practical implementation [1,2,3]. The theoretical security of CV-QKD has been extensively studied in the last 20 years [4,5,6,7,8,9,10,11,12,13,14,15,16], and its practical security has also been demonstrated [17,18,19,20,21,22,23,24,25,26,27,28,29].

In recent years, there has been a growing trend toward CV-QKD networking, and various studies have been conducted to combine it with classical communication systems. One technology that has proven effective in integrating the CV-QKD channel with classical communication is dense wavelength division multiplexing (DWDM). Several experiments have been conducted to demonstrate the feasibility of DWDM on CV-QKD and classical communication, such as the experiments conducted by Bing Qi et al. in 2010 and Rupesh Kumar et al. in 2015 [30,31]. Moreover, in 2018, Fotini Karinou et al. proved that CV-QKD can be combined with existing WDM optical networks [32]. Furthermore, T.A. Eriksson et al. confirmed in 2020 that the CV-QKD channel could be transmitted with 100 WDM channels, achieving a data transmission rate of 18.3 Tb/s [33].

Another technology that can be used to realize the transmission of classical signals and CV-QKD quantum signals is the simultaneous QKD and classical communication (SQCC) protocol. This protocol utilizes the quadrature components of the coherent state to transmit both classical and quantum key information. Bing Qi proposed SQCC in 2016 [34], and simulations were conducted to prove that the protocol can achieve classical communication and CV-QKD with a real local oscillator [35]. In 2017, Tianyi Wang et al. proved through simulations that CV-QKD and classical communication could be achieved with tens of kilometers of fiber transmission under the SQCC protocol [36]. Furthermore, in 2019, Rupesh Kumar et al. conducted experiments to realize SQCC transmission on a 25 km fiber [37].

Reducing the excess noise in CV-QKD, particularly in the presence of classical signals, is an extremely challenging issue. Several technologies have been proposed to address this issue. In 2013, Paul Jouguet et al. proposed the use of larger data blocks to estimate excess noise, which helped resist the data deviation caused by the finite-size effect, allowing for transmission distances exceeding 80 km [38]. In 2015, Duan Huang et al. proposed an accurate phase estimation method, larger data blocks, and higher isolation between the local oscillator (LO) and signal to reduce excess noise [39]. Moreover, the introduction of the local–local oscillator (LLO) CV-QKD scheme [39,40,41] further reduced the photon crosstalk noise caused by LO. The LLO scheme involves using a local LO at the receiver’s side to cancel out the photon crosstalk noise, leading to excess noise reduction and high performance in CV-QKD systems.

However, the coexistence of classical signals with QKD in more complex environments necessitates selecting appropriate channels and maintaining low excess noise levels, posing significant challenges. On the one hand, the excess noise levels in specific channels may exceed the upper limit of excess noise tolerance. On the other hand, the introduction of sudden interference noise may directly impede the transmission of CV-QKD signals through the channel. Unfortunately, effective strategies for selecting suitable transmission channels for CV-QKD have not yet been explored. In order to reduce the impact of burst random high-power channel noise, it is necessary to study the noise-suppression technology in CV-QKD.

In this study, we propose a noise-suppression scheme for CV-QKD based on carrier frequency switching (CFS). This scheme adopts CFS and digital filtering techniques to separate the frequency spectrum of the transmission signal from high-power random noise and effectively filter out most classical noise after coherent detection. To evaluate the excess noise suppression performance of the proposed scheme, we model the channel noise and conduct simulations, analyzing the results from four perspectives: excess noise, secret key rate, transmission distance, and modulation variance. The simulation outcomes indicate that the proposed noise-suppression scheme can effectively mitigate the effects of the high-power channel random noise on the receiver and exhibit stable noise-suppression performances at transmission distances from 5 km to 100 km. Average excess noise values with this noise-suppression scheme can be controlled at $6.05\times 10^{-4}$∼$7.55\times 10^{-4}$. Moreover, the secret key rate is improved and values remain stable at modulation variances from 2 to 22.

The manuscript is organized as follows: Section 2 introduces the proposed CV-QKD noise-suppression scheme based on CFS, including the GG02 protocol and the channel noise model in the CV-QKD system. Section 3 presents the simulations designed to validate and analyze the scheme’s noise suppression performance in terms of the excess noise value, secret key rate, and transmission distance, and discusses the relationship between modulation variance and excess noise. Finally, Section 4 concludes the study.

## 2. Scheme Description

### 2.1. Theoretical GG02 Protocol

**Figure 1:**
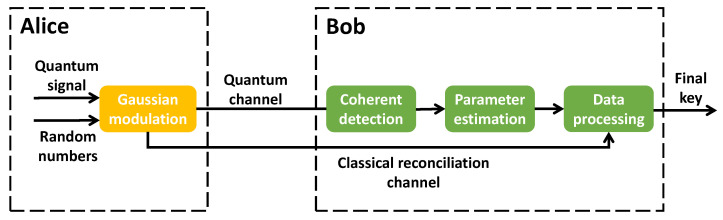
The schematic diagram of the GG02 protocol. Alice prepares quantum signals and transmits them to Bob through the quantum channel. Bob receives transmitted quantum signals and detects the signals’ quadrature components by coherent detection. Data carried by quadrature components are measured by the selected measurement basis. Then the parameter estimation, using part of the data, is conducted to estimate the CV-QKD performance. Finally, data processing is conducted to obtain the final secret key.

CV-QKD, a type of QKD, adopts continuous variables to encode transmission information based on continuously distributed eigenvalues. The GG02 protocol, introduced in 2002, is one well-implemented protocol in CV-QKD. This protocol utilizes the quadrature components of coherent states to transmit the signal, combining Gaussian modulation to facilitate the key distribution between authorized parties [4]. The schematic diagram of the GG02 protocol is depicted in Figure 1, where Alice denotes the transmitter, and Bob represents the receiver. The GG02 protocol involves a series of distinct steps, which are described below.

1. Alice prepares and transmits quantum signals. On the Alice side, she generates two distinct sets of random numbers, $\left\{x_{A}^{j}\right\}$ and $\left\{p_{A}^{j}\right\}$, both of which are distributed according to Gaussian distributions of $N\left(0,V_{A}\right)$. The range of *j* is 0,…,N and *N* represents the number of random numbers. These sets of random numbers are then modulated to quantum signals, resulting in the creation of a coherent state $\left|x_{A}^{j}+ip_{A}^{j}\rangle \right.$ that serves as the transmission quantum signal. Then, she transmits it through the quantum channel to the receiver.
2. Bob receives the quantum signal. Upon receipt of the transmission quantum signal, Bob is responsible for detecting its quadrature components using coherent detection, which can be achieved through either homodyne detection or heterodyne detection. In the case of homodyne detection, Bob must randomly select a measurement basis to measure the coherent signal to obtain one of the quadrature components, either $\left\{x_{B}^{j}\right\}$ or $\left\{p_{B}^{j}\right\}$. Once the measurement is complete, Bob must inform Alice of the adopted measurement basis. Alice only retains the data that are consistent with Bob’s measurement results and discards any irrelevant data. In contrast, if Bob adopts heterodyne detection, both $\left\{x_{B}^{j}\right\}$ and $\left\{p_{B}^{j}\right\}$ are measured simultaneously, obviating the need for measurement basis selection. In this case, Alice retains all of the data.
3. Estimates the CV-QKD system parameters. Alice randomly selects a portion of her data and combines it with the corresponding data from Bob to perform parameter estimation. The key parameters of the system, such as channel transmittance and excess noise, can be calculated using the maximum likelihood estimation algorithm. Based on the parameter estimation results, the secret key rate of the system can be evaluated, ultimately determining the key distribution performance of the system.
4. Data processing. The data that are not used for parameter estimation are employed for the purposes of data reconciliation and privacy amplification. Once these processes are complete, the secret key is obtained.

### 2.2. Channel Noise Model of CV-QKD System

The communication process of CV-QKD can be expressed by a simple linear relationship, as follows:

(1) y=tx+n,

where *y* represents the received signal, *x* represents the transmitted signal, *t* represents the channel transmittance, and *n* represents the channel noise and system intrinsic noise. The noise term *n* is analyzed in order to propose an appropriate noise-suppression scheme that is tailored to the specific noise characteristics of the CV-QKD channel.

Based on the plausibility of the noise source, the noise component in *n* can be categorized into two types: trusted noise and untrusted noise. The trusted noise component, which is primarily comprised of thermal noise and detector shot noise, is generated by legitimate communication parties. Thermal noise is predominantly generated by the thermal motion of electrons in electronic components, such as resistors and metal-oxide-semiconductor field-effect transistors (MOSFETs). The power spectral densities of the resistor’s thermal noise in the voltage and current models can be expressed as $4k_{B}Tr$ and $4k_{B}T/r$, respectively, where $k_{B}$ represents the Boltzmann constant, *T* represents thermodynamic temperature, and *r* represents the resistance value. Similarly, the power spectral density of the MOSFET thermal noise can be expressed as $4k_{B}T\gamma g_{m}$, where γ represents the noise factor and $g_{m}$ represents transconductance. When the temperature, resistance value, and MOSFET parameters remain constant, the power spectral densities of thermal noise generated by the resistor and MOSFET are constant and exhibit characteristics of Gaussian white noise. Shot noise, on the other hand, is caused by quantum light field fluctuations and has a power spectral density expressed as $2e\bar{I}$, where *e* represents the elementary charge and $\bar{I}$ represents the average current in the electronic components. In a stable operation, the power spectral density of shot noise is also constant and white.

Untrusted noise is characterized as noise generated from untrusted sources, including eavesdroppers and other signals co-transmitted with the quantum signal. Since this part of the noise cannot be accurately calibrated, it is considered unreliable. In contrast to trusted noise, which exhibits a white spectrum and constant power spectrum densities, untrusted random noise is characterized by random power, frequency location, and appearance time. This noise can lead to severe interference for the quantum signal, resulting in inaccurate reception at the receiver and performance deterioration of the CV-QKD system. Therefore, it is imperative to suppress untrusted random noise, which is the primary noise type. Additionally, the noise term *n* contains an interference component, which exhibits randomness and similar characteristics to untrusted random noise and, thus, can be considered as untrusted random noise. Due to the limited bandwidth resource, there will be cases where classical signals and quantum signals are co-transmitted at different sidebands in the same wavelength. In this case, high-power random crosstalk will be introduced by co-transmitted classical signals in the quantum channel. Our noise-suppression scheme is proposed to overcome this kind of noise.

The noise model can be established based on the above analysis, as shown in Equation (2).

(2) $$n=n_{\mathrm{eve}}+n_{c}+n_{i}+n_{\mathrm{shot}}+n_{\mathrm{the}}.$$

The notation used in the equation is as follows: $n_{\mathrm{eve}}$ denotes the excess noise arising from eavesdropping by an eavesdropper; $n_{c}$ represents crosstalk due to the presence of a classical signal; $n_{i}$ denotes noise terms that occur randomly and have center frequencies in random frequency bands; $n_{\mathrm{shot}}$ represents shot noise, while $n_{\mathrm{the}}$ represents thermal noise.

**Figure 2:**
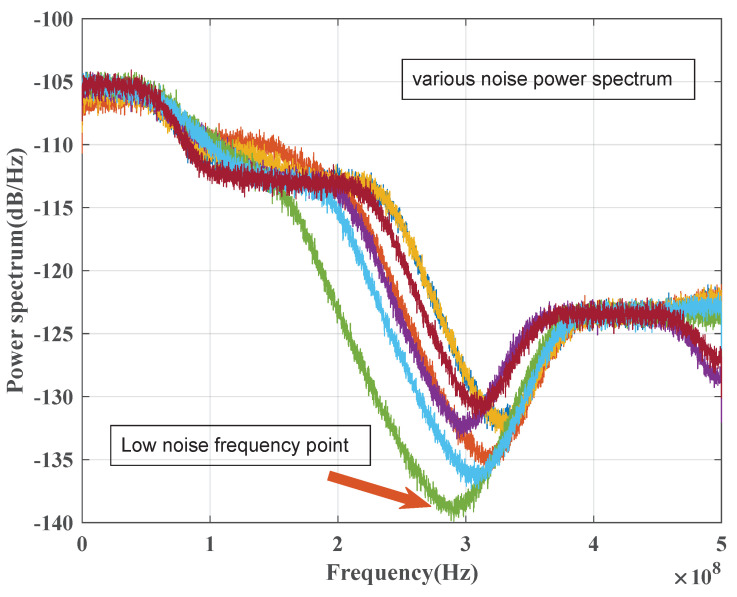
The simulation result of the CV-QKD channel noise power spectrum. The CV-QKD channel noise in this simulation contains crosstalk noise, random interference noise, shot noise, and thermal noise. The simulation is repeated 7 times.

Drawing on the noise model described below, we developed a noise simulation system to simulate the noise in CV-QKD. We simulate a classic baseband communication signal occupying a bandwidth of 100 MHz, and the power is greater than the QKD signal power. This section will cause crosstalk to the QKD signal. In addition, we randomly introduce an interference signal during each communication simulation process, which occurs in a random frequency band and has random power, forming a random interference noise. In addition, we also simulate the stationary Gaussian noise, including the shot noise and thermal noise. Figure 2 presents the simulation results of the CV-QKD noise power spectrum, which includes crosstalk noise, random interference noise, shot noise, and thermal noise. The 7 curves represent that the simulation is repeated 7 times. The simulation demonstrates that the channel noise exhibits colored noise distribution, with significant random fluctuations.

### 2.3. CV-QKD Noise-Suppression Scheme Based on CFS

The analysis presented in Section 2.2 and the simulation results depicted in Figure 2 reveal the presence of substantial fluctuations in the channel of the CV-QKD system due to random noise, which may manifest as high-power noises appearing at any arbitrary frequency band. This poses a challenge for fixed-frequency transmission modulation, as it may be susceptible to severe performance degradation due to such random noise. Therefore, a noise suppression scheme has been proposed to mitigate these effects by separating the transmission signal from the channel noise prior to signal transmission and minimizing the channel noise after the detection. To this end, the noise suppression scheme of the CV-QKD system is designed to achieve two main objectives.

1. Switching the frequency to the minimum noise transmission window. In order to maximize the separation of the transmission signal from high-power channel noise, the frequency switching method is employed to preprocess the signal prior to channel transmission. The channel noise frequency spectrum in different frequency bands exhibits significant fluctuations with varying noise power. The minimum noise transmission window, or “window” for brevity, corresponds to the frequency band with the lowest noise power in the entire channel noise frequency spectrum. By shifting the frequency spectrum of the transmission signal to align with the window, the transmission signal can be effectively separated from high-power channel noise, thereby reducing noise interference on the signal.
2. Digital filtering processing. Despite the use of frequency switching to separate the transmission signal from high-power channel noise, residual noise can still enter the receiver and cause increased excess noise. To minimize this effect, a digital filtering approach can be implemented to allow only the transmission signal and a small amount of channel noise at the same frequency band to pass through, while filtering out high-power noise at other frequency bands. The resulting filtered signal contains fewer noise components, which can facilitate subsequent signal-processing steps at the receiver.

**Figure 3:**
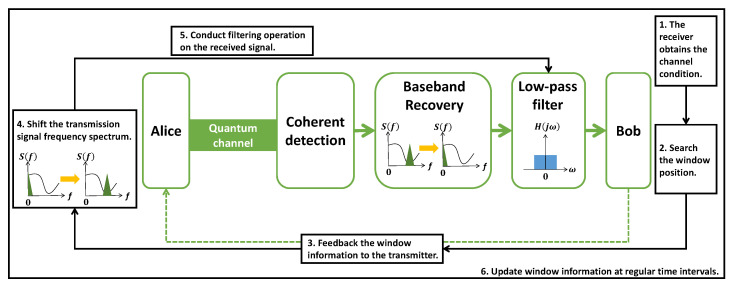
The schematic diagram of the CV-QKD noise-suppression scheme based on CFS. (1) The receiver obtains the channel condition. The power spectrum of channel noise is obtained. (2) The system searches for the window position. The window position is located at the minimum power value. (3) The window information is fed back to the transmitter. (4) The frequency spectrum of the transmission signal is shifted. (5) A filtering operation is conducted on the received signal. Before the filtering operation, the transmission signal frequency spectrum is supposed to move back to the baseband. (6) The window information is updated at regular time intervals. The channel conditions change in real time. Updating the window information in a timely manner can guarantee the scheme’s performance.

By combining the aforementioned steps, we propose a CV-QKD noise-suppression scheme based on CFS. The schematic diagram of this scheme is presented in Figure 3. The following steps outline the proposed approach in detail.

**Figure 4:**
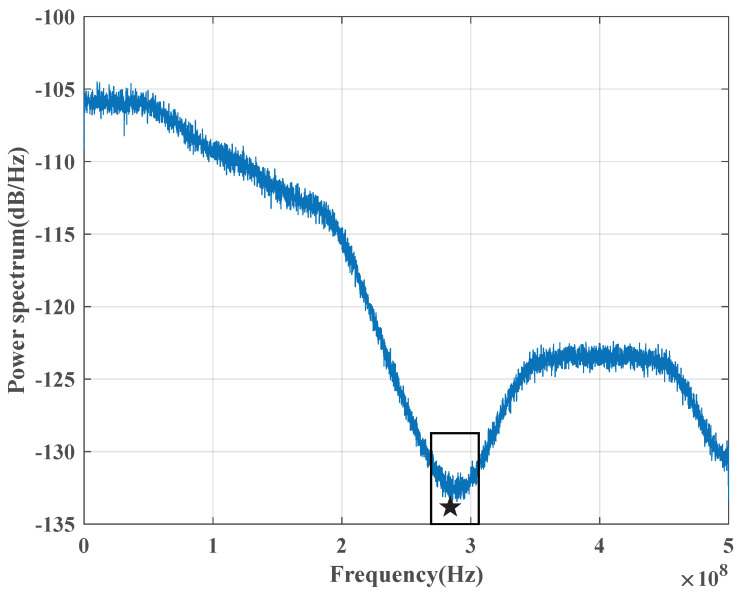
The channel noise power spectrum of the CV-QKD system. The power spectrum has high-power parts and low-power parts. The black star represents the minimum power value of this curve. The frequency band in the black square is the window position.

**Figure 5:**
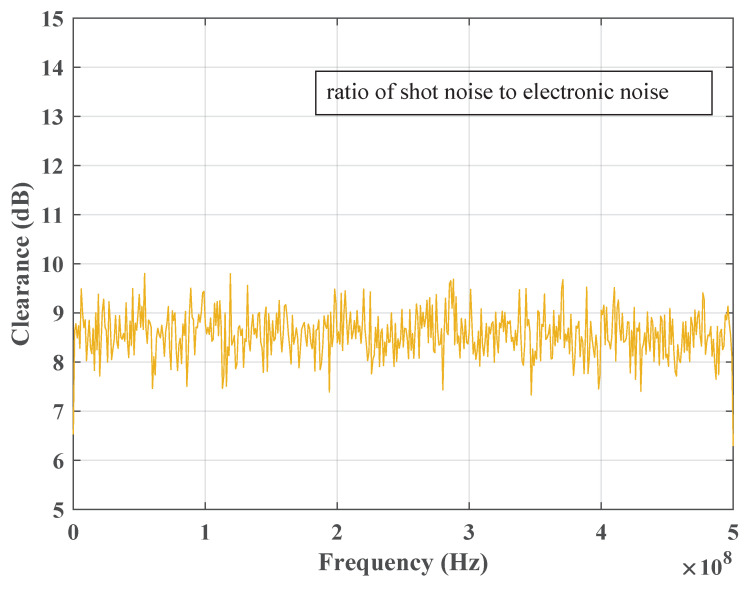
The clearance between electronic noise and shot noise in different frequencies. Since both the shot noise and electronic noise are Gaussian stationary noise, their clearance is also stable, which is consistent with reality.

1. The receiver obtains the channel condition. In order to accurately locate the minimum noise transmission window in the channel noise frequency spectrum, the receiver must first estimate the CV-QKD channel condition, obtain the channel noise frequency spectrum, and compute its power spectrum. This information is then used to determine the correct window position in the subsequent step.
2. Search the window position. Once the power spectrum of the channel noise has been obtained, the position of the minimum power value corresponds to the location of the minimum noise transmission window. Figure 4 depicts the channel noise power spectrum, with the blue curve representing the power spectrum and the black square indicating the window position. The black star on the power spectrum curve indicates the position of the minimum noise transmission window. Figure 5 shows the variation of clearance with frequency.
3. Feedback the window information to the transmitter. After obtaining the window information, the receiver sends it to the transmitter for signal modulation.
4. Shift the transmission signal frequency spectrum. Upon obtaining the window information, the transmitter subsequently shifts the frequency spectrum of the transmission signal from the baseband to the carrier frequency. The resulting transmission signal is then transmitted through the quantum channel for further processing.
5. Conduct the filtering operation on the received signal. Once the signal has been received by the receiver, it is necessary to shift its center frequency from the window position to the baseband. This is followed by a digital low-pass filtering operation, which removes high-power noise outside the baseband while allowing the transmission signal and a minimal amount of channel noise to pass through. The signal that passes through the low-pass filter is subsequently used for data processing. By adopting a Butterworth low-pass filter in this stage, the noise components can be effectively filtered out. The amplitude characteristic of this filter can be expressed by Equation (3).
  (3) $${\left|H\left(j\omega \right)\right|}^{2}=\frac{1}{1+{\left(\frac{\omega }{\omega_{c}}\right)}^{2N}},$$
  where $\left|H\left(j\omega \right)\right|$ denotes the amplitude characteristic of the filter, while $\omega_{c}$ and *N* correspond to the cut-off frequency and the filter order, respectively. The Butterworth filter is selected for its flat spectrum, which results in minimal additional interferences. Thus, in the proposed noise-suppression scheme, a Butterworth low-pass filter is utilized to process the signals.
6. Update the window information at regular time intervals. As the channel condition is subject to random fluctuations, the window position in the channel noise power spectrum may also change. To prevent performance deterioration of the CV-QKD system due to signal interference, it is necessary to update the window position periodically instead of relying on the initially searched position. The specific time interval for updating can be determined according to the practical requirements and system characteristics. For conventional slowly changing channels, updates of the channel conditions can be done hourly. For channels that change quickly, channel monitoring can be performed every minute.

## 3. Performance Analysis and Verification

To assess the effectiveness of the suggested noise-suppression scheme, we devised corresponding simulations. To properly represent the characteristics of the scheme, we conducted experiments by establishing an experimental group and a control group and comparing the simulation results of the two groups. The experimental group employed the proposed noise-suppression scheme to process the signal, which moved the frequency spectrum to the dynamically changing carrier frequency based on feedback. In contrast, the control group did not search the window and instead shifted the frequency spectrum to a fixed frequency. All other parameters were identical for the two groups. The effectiveness of the proposed approach was evaluated based on multiple criteria, including excess noise, the secret key rate, transmission distance, and modulation variance. In order to reduce randomness in the simulations, Monte Carlo simulations were conducted, repeating simulations several times and calculating their mathematical statistics.

### 3.1. Excess Noise at the Same Transmission Distance

Excess noise is a crucial metric in CV-QKD, as it directly impacts system performance. Lower excess noise values are indicative of improving CV-QKD system performance. In this subsection, we compare the excess noise values of the experimental group and two control groups, while maintaining the same transmission distance, to evaluate the performance of the proposed noise-suppression scheme.

**Figure 6:**
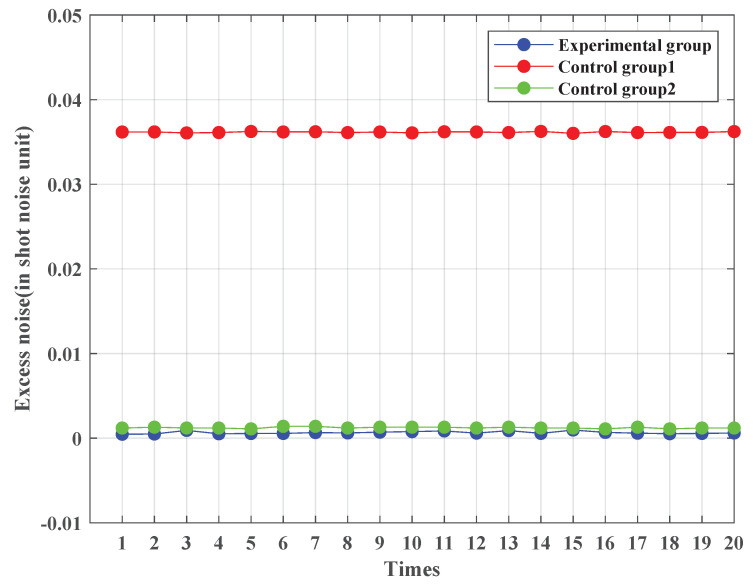
Excess noise values of the experimental and two control groups (no channel attenuation). The length of the transmission signal is set to $1\times 10^{5}$. The symbol rate is set to 10 MHz. The sampling frequency is set to 1 GHz. The sampling pulse is a raised-cosine pulse. The filter type is the Butterworth low-pass filter. The excess noise calculation is repeated 20 times for both experimental and control groups. For control group 1, the excess noise’s mathematical expectation is $3.62\times 10^{-2}$ and its standard deviation is $6.69\times 10^{-5}$. For control group 2, the excess noise’s mathematical expectation is $1.20\times 10^{-3}$ and its standard deviation is $8.75\times 10^{-5}$. For the experimental group, the excess noise’s mathematical expectation is $6.57\times 10^{-4}$ and its standard deviation is $1.45\times 10^{-4}$.

For this simulation, the transmission signal length is $1\times 10^{5}$, the symbol rate is 10 MHz, the sampling frequency is 1 GHz, and a raised-cosine pulse is employed as the sampling pulse. In future practical implementations, we will also consider root-raised cosine filters to further ensure signal integrity and noise suppression. The Butterworth filter used in the simulation has a normalized pass-band edge frequency of 0.2 and a stop-band edge frequency of 0.4, with maximum pass-band attenuation and minimum stop-band attenuation set to 1 dB and 10 dB, respectively. To simulate the channel noise in CV-QKD, active base-band noise, multiple frequency-band noises, and shot noise are added. The experimental and two control groups are all subjected to 20 iterations. The transmission signal frequency spectrum of control group 1 is shifted to 100 MHz. The transmission signal frequency spectrum of control group 2 is shifted to 300 MHz. The quantum efficiency is 0.6, the modulation variance is 2, and the electrical noise is 0.1. When the transmission distance and fiber transmission loss are not considered, the excess noise calculation results of the experimental and control groups are depicted in Figure 6. The abscissa represents the calculation time, while the ordinate represents the excess noise value. The simulation result shows that the excess noise value range of the 20 experimental groups is $4.69\times 10^{-4}$∼$9.60\times 10^{-4}$, the excess noise value range of the 20 first control groups is $3.60\times 10^{-2}$∼$3.63\times 10^{-2}$, and the excess noise value range of the 20 second control groups is $1.10\times 10^{-3}$∼$1.40\times 10^{-3}$. As demonstrated by the results, the excess noise value of the experimental group is significantly lower than that of the control group. Its excess noise value is about 1.8% of that under the conventional scheme.

**Figure 7:**
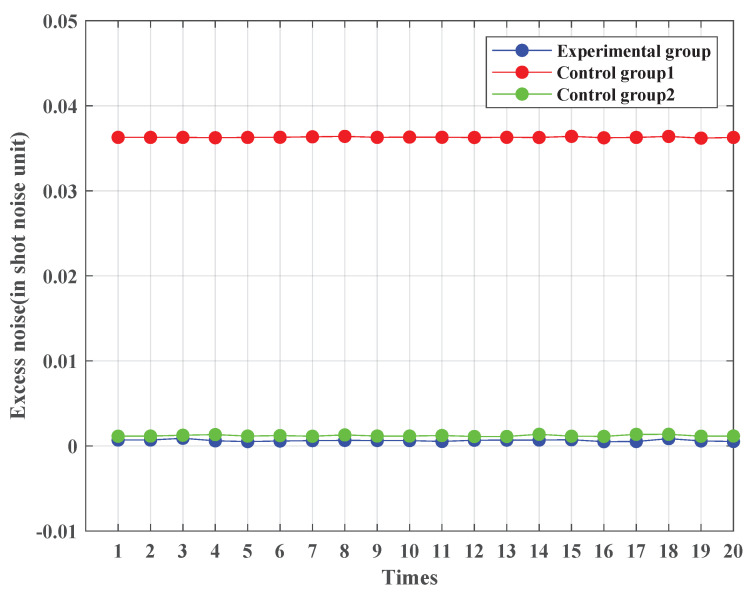
Excess noise values of the experimental and two control groups (5 km fiber attenuation). The length of the transmission signal is set to $1\times 10^{5}$. The symbol rate is set to 10 MHz. The sampling frequency is set to 1 GHz. The sampling pulse is a raised-cosine pulse. The transmission distance is set to 5 km. The fiber transmission loss is set to 0.2 dB/km. The filter type is the Butterworth low-pass filter. The excess noise calculation is repeated 20 times for both experimental and control groups. For control group 1, the excess noise’s mathematical expectation is $5.26\times 10^{-2}$ and its standard deviation is $5.48\times 10^{-5}$. For control group 2, the excess noise’s mathematical expectation is $1.20\times 10^{-3}$ and its standard deviation is $9.00\times 10^{-5}$. For the experimental group, the excess noise’s mathematical expectation is $6.44\times 10^{-4}$ and its standard deviation is $1.05\times 10^{-4}$.

To better emulate practical fiber transmission conditions, it is necessary to incorporate transmission distance and fiber transmission loss in the simulation. In this simulation, a fiber transmission loss of 0.2 dB/km is set, and transmission is carried out over 5 km. Other parameters are kept unchanged. Figure 7 displays the excess noise calculation results of the experimental and two control groups over a 5 km fiber transmission. The simulation result shows that the excess noise value range of the 20 experimental groups is $5.06\times 10^{-4}$∼$9.05\times 10^{-4}$, the excess noise value range of the 20 first control groups is $3.62\times 10^{-2}$∼$3.64\times 10^{-2}$, and the excess noise value range of the 20 second control groups is $1.11\times 10^{-3}$∼$1.37\times 10^{-3}$. The results reveal that the excess noise value of the experimental group remains significantly lower than that of the two control groups. These findings demonstrate that the noise-suppression scheme based on CFS is capable of effectively mitigating the impact of the channel noise on the receiver in a CV-QKD system.

### 3.2. Secret Key Rate at the Same Transmission Distance

**Figure 8:**
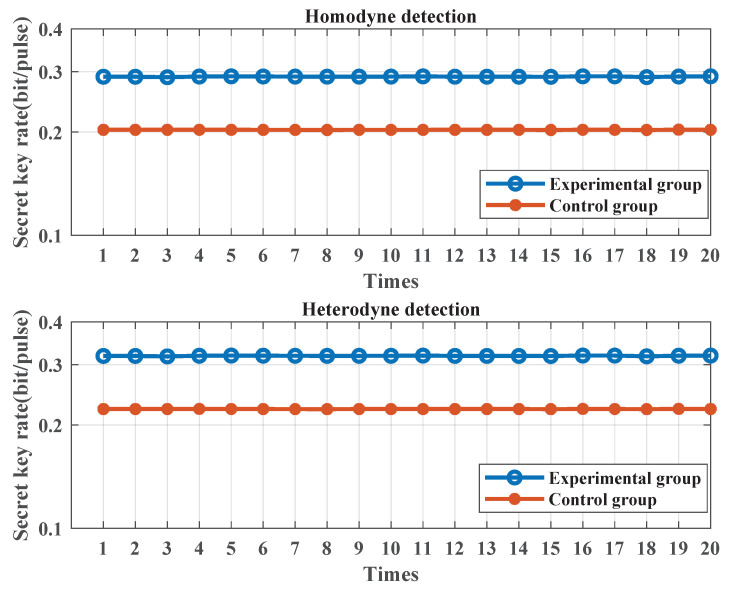
Secret key rate values of the experimental and control groups (5 km fiber attenuation). The length of the transmission signal is set to $1\times 10^{5}$ and the modulation variance is set to 4. The symbol rate is set to 10 MHz. The sampling frequency is set to 1 GHz. The sampling pulse is a raised-cosine pulse. The transmission distance is set to 5 km. The fiber transmission loss is set to 0.2 dB/km. The filter type is the Butterworth low-pass filter. The secret key rate is repeatedly calculated 20 times for both experimental and control groups.

In this subsection, the secret key rates of the experimental and control groups are, respectively, calculated at the same transmission distance. One can see Appendix A for the calculating formula of the secret key rate. For this simulation, the transmission distance is set to 5 km, with a fiber transmission loss of 0.2 dB/km. The transmission signal frequency spectrum of the control group is shifted to 100 MHz. Other parameters remain the same as those in Section 3.1. Figure 8 depicts the secret key rate calculation results of the experimental and control groups over a 5 km fiber transmission. Key rates of the two groups are calculated under both homodyne and heterodyne detection. As evidenced by the simulation results, the secret key rate of the experimental group, under both homodyne and heterodyne detection, is higher than that of the control group. Although there is only one comparison, the proposed scheme has optimal performance according to the lower excess noise in Section 3.1. These findings demonstrate that the proposed scheme is capable of enhancing the performance of a CV-QKD system.

### 3.3. Secret Key Rate at Different Transmission Distances

**Figure 9:**
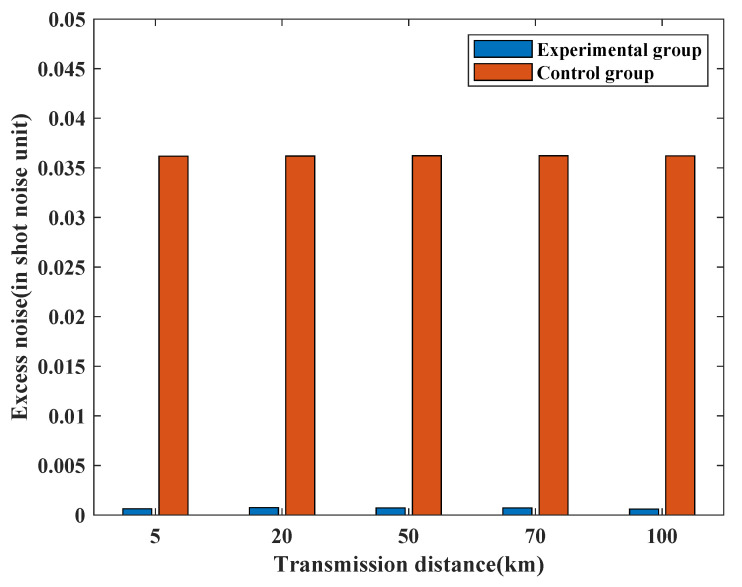
Excess noise mathematical expectation values of the experimental group and the control group at different transmission distances. The length of the transmission signal is set to $1\times 10^{5}$. The symbol rate is set to 10 MHz. The sampling frequency is set to 1 GHz. The sampling pulse is a raised-cosine pulse. The filter type is the Butterworth low-pass filter. Transmission distances are set to 5 km, 20 km, 50 km, 70 km, and 100 km. The fiber transmission loss is set to 0.2 dB/km. The Monte Carlo simulation is conducted for statistical excess noise mathematical expectations at different transmission distances. The excess noise calculation is repeated 10 times for both experimental and control groups at each transmission distance and its mathematical expectation is calculated.

Taking into consideration the fiber transmission loss, the quantum signal’s reception is affected as the channel transmission distance increases, leading to greater loss. In this subsection, we compare the excess noises of the experimental and control groups at different transmission distances. For this simulation, five transmission distances are set: 5 km, 20 km, 50 km, 70 km, and 100 km, respectively, with a fiber transmission loss of 0.2 dB/km. The transmission signal frequency spectrum of the control group is shifted to 100 MHz. To reduce randomness, we calculate the excess noise of each group at each transmission distance 10 times and take their mathematical expectation. Other parameters remain the same as those set in Section 3.1. Figure 9 depicts the mathematical expectations of the experimental and control groups at different transmission distances.

The simulation results indicate that the excess noise mathematical expectation value of the experimental group is significantly smaller than that of the control group at every transmission distance, and remains relatively stable. Specifically, at transmission distances of 5 km, 20 km, 50 km, 70 km, and 100 km, the excess noises of the experimental groups are $6.35\times 10^{-4}$, $7.55\times 10^{-4}$, $7.32\times 10^{-4}$, $7.32\times 10^{-4}$, and $6.05\times 10^{-4}$, respectively, while those of the control groups are $3.62\times 10^{-2}$, $3.62\times 10^{-2}$, $3.62\times 10^{-2}$, $3.62\times 10^{-2}$, and $3.62\times 10^{-2}$, respectively. The excess noise values of the experimental groups are two orders of magnitude smaller than those of the control groups, and remain numerically stable. These findings suggest that the proposed noise-suppression scheme exhibits good noise-suppression capabilities and stable performance across different transmission distances.

**Figure 10:**
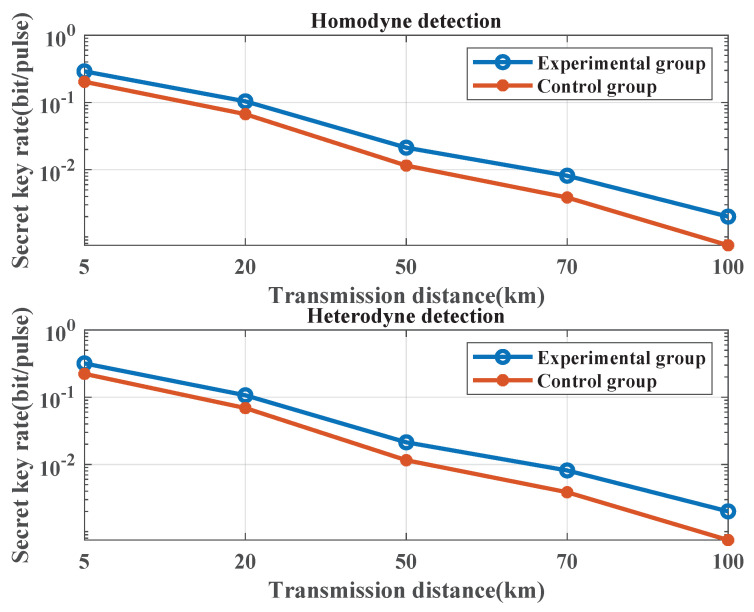
Excess noise mathematical expectation values of the experimental group and the control group at different transmission distances. The length of the transmission signal is set to $1\times 10^{5}$ and the modulation variance is set to 4. The symbol rate is set to 10 MHz. The sampling frequency is set to 1 GHz. The sampling pulse is a raised-cosine pulse. The filter type is the Butterworth low-pass filter. Transmission distances are set to 5 km, 20 km, 50 km, 70 km, and 100 km. The fiber transmission loss is set to 0.2 dB/km. Excess noise values used to calculate secret key rates are the mathematical expectations shown in Figure 9.

The excess noise, obtained from a simulation comprising 5 experimental groups and 5 control groups, is used to calculate secret key rate values using homodyne and heterodyne detection methods. The calculation formula for the secret key rate is provided in Appendix A. Figure 10 illustrates the secret key rates of experimental and control groups at varying transmission distances. The simulation results indicate that, under both homodyne and heterodyne detection, the secret key rates of experimental groups surpass those of control groups at different transmission distances. As the transmission distance increases, the secret key rates of both experimental and control groups decrease. However, secret key rate values of experimental groups are always higher than that of control groups. With the increase of the transmission distance, secret key rates of 5 experimental groups are 1.43, 1.55, 1.85, 2.08, and 2.86 times that of the control groups, respectively, which indicates that the proposed noise-suppression scheme can improve the CV-QKD performance at different transmission distances.

### 3.4. Secret Key Rate at Different Modulation Variances

In this subsection, we calculate the secret key rates of the experimental and control groups with different modulation variances to provide guidance for determining the optimal modulation variance at the transmitter when the proposed scheme is used in practice. The modulation variance is set as a variable, and its values are 2, 6, 10, 14, 18, 22, 26, 30, 34, and 38, respectively. The transmission signal frequency spectrum of the control group is shifted to 100 MHz. To reduce the effect of random errors, we calculate the secret key rates of both groups 5 times and compute their mathematical expectations. The transmission distance is set to 5 km, and the fiber transmission loss is set to 0.2 dB/km. All other parameters are consistent with those in Section 3.1.

**Figure 11:**
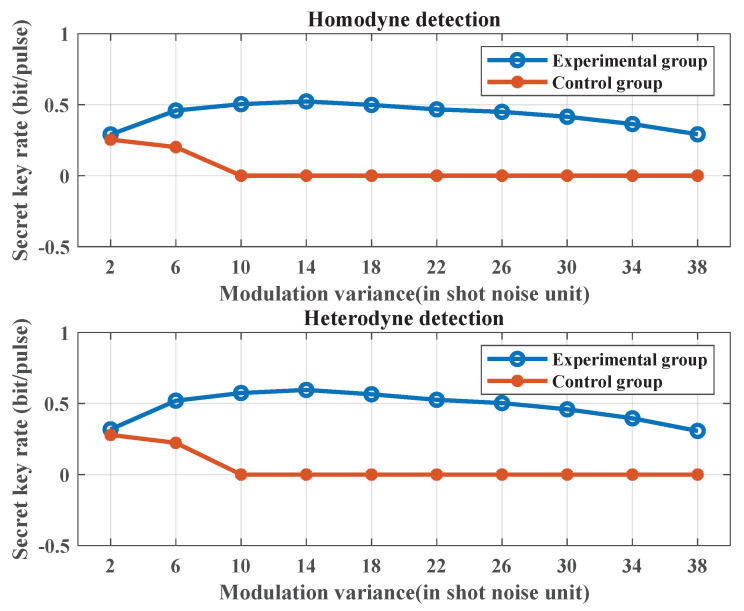
Secret key rate mathematical expectation values of the experimental and control groups at different modulation variances. The length of the transmission signal is set to $1\times 10^{5}$. The symbol rate is set to 10 MHz. The sampling frequency is set to 1 GHz. The sampling pulse is a raised-cosine pulse. The filter type is the Butterworth low-pass filter. Transmission distances are set to 5 km. The fiber transmission loss is set to 0.2 dB/km. Modulation variances are set to 2, 6, 10, 14, 18, 22, 26, 30, 34, and 38. The Monte Carlo simulation is conducted for the statistical secret key rate mathematical expectations with different modulation variances. The secret key rate is repeatedly calculated 5 times for both experimental and control groups with each modulation variance and mathematical expectation calculated.

The figure presented in this section, Figure 11, depicts the mathematical expectation values of the secret key rates for the experimental and control groups with different modulation variances under homodyne and heterodyne detections, respectively. The calculation results demonstrate that the secret key rates of the experimental and control groups are close when the modulation variance is set to 2 under both detection methods, with the experimental group having a slightly higher value. However, with the increasing modulation variance, the experimental group, adopting the CFS scheme, can generate the secret key, while the control group, adopting the traditional scheme, can only generate a secret key with smaller modulation variances. This is because the random noise power may be high at the modulation frequency in the control group, making it difficult to generate the secret key after averaging several repeated simulations. The experimental group can correctly find the minimum noise window position each time and can generate the secret key with different modulation variances after several repeated simulations. The simulation result shows that the proposed scheme is robust with different modulation variances.

## 4. Conclusions

In this paper, a novel noise-suppression scheme for CV-QKD based on CFS is proposed. The scheme dynamically searches for the minimum-power window of the channel noise power spectrum in CV-QKD and adopts the CFS method to shift the frequency spectrum to the window to avoid high-power noise. Digital filtering is then used to reduce the channel noise entering the receiver as much as possible. Controlled simulations are conducted to verify the scheme’s performance in terms of excess noise, secret key rate, transmission distance, and modulation variance. Simulation results demonstrate that the noise-suppression scheme can effectively suppress channel noise and significantly improve the CV-QKD performance, with good and stable performance.

The proposed scheme can be combined with synchronization technology at the receiver end to improve the synchronization success rate and achieve stable CV-QKD. Moreover, this scheme has broad applications in scenarios such as simultaneous quantum–classical channel transmission and quantum networks, providing a reliable solution to resist high-power random channel noise.

## Data Availability

The data that support the findings of this study are available from the corresponding author upon reasonable request.

## Abbreviations

The following abbreviations are used in this manuscript:

CFS	carrier frequency switching
QKD	quantum key distribution
CV-QKD	continuous-variable quantum key distribution
DWDM	dense wavelength division multiplexing
SQCC	simultaneous QKD and classical communication
LO	local oscillator
LLO	local–local oscillator
MOSFET	metal-oxide-semiconductor field-effect transistor

## Appendix A. Calculation of the Secret Key Rate

Here, we present the secret key rate calculation process in the asymptotic case. Firstly, the secret key rate for reverse reconciliation is calculated as [42]

(A1) $$R=\beta I_{AB}-\chi_{BE},$$

where β∈(0,1) is the efficiency of reverse reconciliation, $I_{AB}$ is the mutual information between Alice and Bob, and $\chi_{BE}$ is the maximum information available to Eve on Bob’s key, bounded by the Holevo quantity. Specifically, $I_{AB}^{\mathrm{hom}}$, $I_{AB}$ with homodyne detection, can be identified as

(A2) $$I_{AB}^{\mathrm{hom}}=\frac{1}{2}\log_{2}\frac{V+\chi_{\mathrm{tot}}}{1+\chi_{\mathrm{tot}}},$$

$I_{AB}^{\mathrm{het}}$, $I_{AB}$ with heterodyne detection, can be identified as

(A3) $$I_{AB}^{\mathrm{het}}=\log_{2}\frac{V+\chi_{\mathrm{tot}}}{1+\chi_{\mathrm{tot}}},$$

where $V=V_{A}+1$, $\chi_{\mathrm{tot}}$ represents the total noise that is referred to the channel input. $V_{A}$ represents the modulation variance. Moreover, $\chi_{BE}$ is identified as follows:

(A4) $$\begin{array}{c} \chi_{BE}=\sum_{i=1}^{2}G\left(\frac{\lambda_{i}-1}{2}\right)-\sum_{i=3}^{5}G\left(\frac{\lambda_{i}-1}{2}\right), \end{array}$$

where $G\left(x\right)=\left(x+1\right)\log_{2}\left(x+1\right)-x\log_{2}x$. $\lambda_{i}$ are symplectic eigenvalues derived from the covariance matrices and can be expressed as

(A5) $$\begin{array}{ccc} \lambda_{1,2}^{2} & = & \frac{1}{2}\left(A\pm \sqrt{A^{2}-4B}\right),\\ \lambda_{3,4}^{2} & = & \frac{1}{2}\left(C\pm \sqrt{C^{2}-4D}\right),\\ \lambda_{5} & = & 1, \end{array}$$

where

(A6) $$\begin{array}{ccc} A & = & V^{2}\left(1-2T\right)+2T+T^{2}{\left(V+\chi_{\mathrm{line}}\right)}^{2},\\ B & = & T^{2}{\left(V\chi_{\mathrm{line}}+1\right)}^{2}. \end{array}$$

*C* and *D* have different methods for taking values under homodyne detection and heterodyne detection. $C_{hom}$ and $D_{hom}$ are values under homodyne detection, whose expressions are shown in Equation (A7). $C_{het}$ and $D_{het}$ are values under homodyne detection, whose expressions are shown in Equation (A8).

(A7) $$\begin{array}{ccc} C_{hom} & = & \frac{A\chi_{\mathrm{hom}}+V\sqrt{B}+T\left(V+\chi_{\mathrm{line}}\right)}{T(V+\chi_{\mathrm{tot}})},\\ D_{hom} & = & \frac{V\sqrt{B}+B\chi_{\mathrm{tot}}}{T(V+\chi_{\mathrm{tot}})}. \end{array}$$

(A8) $$\begin{array}{ccc} C_{het} & = & \frac{1}{{\left(T\left(V+\chi_{\mathrm{tot}}\right)\right)}^{2}}[A\chi_{\mathrm{het}}^{2}+B+1\\ & + & 2\chi_{\mathrm{het}}\left(V\sqrt{B}+T\left(V+\chi_{\mathrm{line}}\right)\right)+2T\left(V^{2}-1\right)],\\ D_{het} & = & {\left(\frac{V+\sqrt{B}\chi_{\mathrm{het}}}{T(V+\chi_{\mathrm{tot}})}\right)}^{2}. \end{array}$$

In the above formula, $\chi_{\mathrm{line}}=1/T-1+\varepsilon$, $\chi_{\mathrm{hom}}=\left[1+\left(1-\eta \right)+v_{el}\right]/\eta$, and $\chi_{\mathrm{het}}=\left[1+\left(1-\eta \right)+2v_{el}\right]/\eta$. *T* represents channel transmittance. ε represents excess noise. $v_{el}$ represents electrical noise. η represents the quantum efficiency. ε has a relationship with *n* in Equation (2):

(A9) $$\varepsilon =\frac{n-n_{shot}-n_{the}}{T\eta }$$

where $n_{shot}$ represents the shot noise and $n_{the}$ represents the thermal noise.

According to the above calculation formula, the secret key rate is eventually evaluated.
